# The Treatment of Complex Coronary Artery Disease

**DOI:** 10.31083/RCM46214

**Published:** 2025-11-13

**Authors:** Arja Suzanne Vink, Marcel A.M. Beijk

**Affiliations:** ^1^Department of Cardiology, Amsterdam University Medical Center, University of Amsterdam, 1105 AZ Amsterdam, The Netherlands

Coronary artery disease (CAD) is a leading cause of morbidity and mortality worldwide despite the remarkable advances in the prevention, diagnosis and treatment of cardiovascular disease [1]. In recent years, the growing elderly population has led to a marked increase in the prevalence of multivessel disease and complex coronary stenoses, driven by greater atherosclerotic plaque burden, vascular calcification, and a higher incidence of comorbidities. Although a uniformly adopted definition for complex CAD as well as complex percutaneous coronary intervention (PCI) is lacking, it can be referred to as patients with multivessel disease, left main coronary artery disease, chronic total occlusion (CTO), diffuse coronary artery disease with long lesions, ostial lesions, severely calcified lesions, fibrocalcific or undilatable lesions, bifurcation or trifurcation disease, ST-elevation myocardial infarction (STEMI), degenerated saphenous vein graft lesions, or (multiple) comorbidities that may increase risk for adverse events [2]. Another contributing factor is the proportion of very elderly patients who have been turned down by the Heart Team for surgical revascularization [3].

Patients with complex CAD, particularly older adults, often present with atypical angina symptoms such as shortness of breath, fatigue, dizziness, or even syncope, rather than classic chest pain. The most common symptom is a noticeable limitation in exercise capacity. This may manifest as early fatigue, breathlessness on minimal exertion, or the inability to perform previously tolerated physical activities. These limitations are often underrecognized but can be key early signs of underlying myocardial ischemia or left ventricular dysfunction.

Diagnostic evaluation to accurately assess the severity of lesions and myocardial ischemia in complex CAD remains challenging. Non-invasive imaging modalities (Table 1), including computed tomography (CT) coronary angiography (CTCA), stress echocardiography, single-photon emission computed tomography (SPECT), positron emission tomography (PET), and cardiac magnetic resonance (CMR), each have strengths and limitations. CTCA provides excellent anatomic detail but is limited by calcification artifacts in heavily diseased vessels [4]. Functional imaging techniques such as SPECT and stress echocardiography can detect ischemia but may underestimate its extent in multivessel disease due to balanced ischemia. PET and stress CMR offer superior spatial resolution and quantitative perfusion assessment, improving detection of subtle ischemia [5]. However, these non-invasive approaches often require complementary invasive physiological assessment, such as fractional flow reserve (FFR) or instantaneous wave-free ratio (iFR), particularly in patients with diffuse disease, CTO, or left main involvement, to effectively guide revascularization decisions.

**Table 1.** **Comparison table: non-invasive imaging in complex CAD**.

Modality	Strengths	Limitations	Role in complex CAD
Computed tomography (CT) coronary angiography (CTCA)	Excellent anatomic resolution; detects plaques	Limited by heavy calcification and motion artifacts	Useful for anatomy; combine with FFR-CT for physiology
SPECT	Widely available; detects regional ischemia	Limited spatial resolution; risk of balanced ischemia	Screening; may miss multivessel disease
PET	Quantitative perfusion; high sensitivity	Limited availability; radiation exposure	Best for microvascular & multivessel assessment
Stress echocardiography	No radiation; bedside availability	Operator dependent; limited in poor acoustic windows	Good for initial functional evaluation
Stress CMR	High spatial resolution; tissue characterization	Contraindicated in some implants; availability	Excellent for perfusion & viability assessment

CAD, coronary artery disease; SPECT, single-photon emission computed tomography; PET, positron emission tomography; CMR, cardiac magnetic resonance; FFR, fractional flow reserve.

Coronary angiography remains the cornerstone for anatomical assessment, providing detailed visualization of lesion morphology, vessel tortuosity, presence of calcification, and chronic total occlusions (CTOs) [6]. However, angiography alone often underestimates the functional significance of intermediate lesions, particularly in diffuse or multivessel disease. Intracoronary physiology assessments (Table 2), including fractional flow reserve (FFR), and non-hyperemic pressure ratios (i.e., instantaneous wave-free ratio (iFR), resting full-cycle ratio (RFR), diastolic pressure ratio (dPR), and Pd/Pa (whole cycle)), are essential for identifying flow-limiting lesions and guiding revascularization [7]. Intravascular imaging modalities such as intravascular ultrasound (IVUS) and optical coherence tomography (OCT) offer high-resolution visualization of plaque composition and vessel size enabling precise characterization of lesions [8]. Intracoronary imaging may guide PCI, especially in complex CAD, and can accurately assess stent apposition [9]. Hybrid approaches that combine physiological and imaging data are increasingly advocated for managing complex lesions, including bifurcations, left main disease, and heavily calcified segments.

**Table 2.** **Invasive assessment in complex CAD**.

Technique	Strengths	Limitations	Role in complex CAD
Coronary angiography	Gold standard for anatomy; identifies stenosis, CTOs	No physiological data; 2D projection limits	Initial anatomical assessment
Fractional flow reserve (FFR)	Functional assessment; guides PCI decisions	Requires hyperemia; may be influenced by microvascular disease	Identifies flow-limiting lesions
Non-hyperemic pressure ratios	No hyperemia required; faster assessment	Limited data in microvascular dysfunction	Alternative to FFR for physiology-guided PCI
Intravascular ultrasound (IVUS)	Visualizes vessel size, plaque burden; guides stent sizing	Lower resolution than OCT	Useful in left main disease, CTOs, calcified lesions
Optical coherence tomography (OCT)	High-resolution plaque and stent visualization	Limited penetration; requires blood clearance	Best for stent optimization and plaque morphology
Hybrid (FFR + IVUS/OCT)	Combines functional and anatomical data	Increased procedural complexity, cost	Optimal in bifurcations, diffuse disease

CTOs, chronic total occlusions; PCI, percutaneous coronary intervention.

In complex CAD, therapeutic strategy must be individualized through collaboration with the Heart Team, integrating clinical status, anatomical complexity (e.g., Synergy Between PCI with TAXUS and Cardiac Surgery (SYNTAX) score), comorbidities (e.g., diabetes, left ventricle dysfunction, peripheral disease, prior surgery, advanced age), and patient preferences. The SYNTAX score provides a comprehensive assessment of coronary lesion complexity and aids in determining the optimal revascularization strategy. A high SYNTAX score (≥33) is linked to higher rates of repeat revascularization and worse outcomes with PCI compared to surgical revascularization (CABG) [10]. However, its validation is limited to low-risk patients (mean EuroSCORE 3.8 ± 2.7 in the CABG group), and data on patients with higher levels of comorbidities or complex CAD are lacking. To address these limitations, the SYNTAX II score incorporates clinical variables such as age, renal function, left ventricle ejection fraction, peripheral artery disease, and Chronic Obstructive Pulmonary Disease (COPD) along with the anatomical SYNTAX I score [11]. In complex CAD patients, the SYNTAX II score often predicts similar 4-year mortality risks for CABG and PCI. Ultimately, treatment decisions in CAD rely on Heart Team discussions and patient preference.

PCI in patients with complex CAD does not inherently constitute a complex procedure, yet complex CAD frequently necessitates advanced interventional strategies. These include prolonged procedure times, increased use of contrast agents, and heightened risk of complications—all of which are highly operator-dependent. Complex PCI is often characterized by challenging wire crossing (e.g., calcified or occluded vessels), extensive lesion preparation (rotational atherectomy, cutting/scoring balloon angioplasty, or lithotripsy), and difficulty in device delivery, particularly in heavily calcified arteries. In addition, a higher proportion of procedural complications (e.g. acute vessel occlusion, dissection, perforation, and hemodynamic compromise) can be observed [12]. Although contemporary techniques have improved technical success rates, these complex lesions remain a major driver of repeat revascularization. Moreover, complex PCI in complex CAD has been linked to an increased in-hospital and 1-year mortality compared to simpler interventions [10]. Complex PCI often also implies high-risk PCI and typically involves patients with significant comorbidities such as impaired left ventricular ejection fraction, low cardiac output, or renal dysfunction, often requiring mechanical circulatory support (e.g., Impella or extracorporeal life support (ECMO)) [13]. Many of these patients are elderly, surgical turn-downs, or those for whom the Heart Team or the patient preferred an interventional approach.

The use of large-bore arterial access has become a cornerstone of contemporary interventional cardiology, particularly in the context of complex PCI and the deployment of mechanical circulatory support devices such as Impella or veno-arterial-ECMO. Mechanical circulatory support devices allow operators to perform complex PCI without time constraints when hemodynamic deterioration occurs. In this special issue of Reviews in Cardiovascular Medicine, Dong *et al*. [14] conducted a retrospective study in 76 patients with ischemic cardiomyopathy (iCMP) who underwent ECMO-assisted PCI between 2013 and 2022. The baseline mean left ventricular ejection fraction was 30%, which significantly improved at 6 months to 36% and remained higher than baseline at 12 months. Complete revascularization was achieved in 58% of patients. ECMO-related complications included bleeding (8%) and lower limb ischemia (5%). At 12 months, all-cause mortality was 30% and freedom from major adverse cardiac and cerebrovascular events (MACCEs) was 59%. These findings suggest that ECMO-assisted PCI is feasible and safe in high-risk iCMP patients, with favorable left ventricular remodeling and survival outcomes. Importantly, as device sizes and procedural complexity increase, operator proficiency, multidisciplinary collaboration, and adherence to best practices in vascular access are critical to improving procedural safety and patient outcomes.

Complex PCI is an evolving area in interventional cardiology, but evidence remains limited, and no uniform definition exists. Varying criteria across studies limit comparisons, and no randomized trials have compared complex PCI with CABG or medical therapy, as these patients are often excluded from major trials. Coronary complexity, comorbidities, and operator experience further complicate the development of standardized strategies. The treatment of complex CAD requires individualized assessment and a multidisciplinary approach. Tools such as intracoronary physiology and intravascular imaging can reduce procedural complexity and improve outcomes.

## References

[1] Roth GA, Johnson C, Abajobir A, Abd-Allah F, Abera SF, Abyu G (2017). Global, Regional, and National Burden of Cardiovascular Diseases for 10 Causes, 1990 to 2015. *Journal of the American College of Cardiology*.

[2] Lawton JS, Tamis-Holland JE, Bangalore S, Bates ER, Beckie TM, Bischoff JM (2022). 2021 ACC/AHA/SCAI Guideline for Coronary Artery Revascularization: A Report of the American College of Cardiology/American Heart Association Joint Committee on Clinical Practice Guidelines. *Circulation*.

[3] Landes U, Bental T, Levi A, Assali A, Vaknin-Assa H, Lev EI (2018). Temporal trends in percutaneous coronary interventions thru the drug eluting stent era: Insights from 18,641 procedures performed over 12-year period. *Catheterization and Cardiovascular Interventions: Official Journal of the Society for Cardiac Angiography & Interventions*.

[4] Seitun S, Mantini C, Clemente A, Sambuceti V, Francese G, Carpaneto S (2025). Role of CT and CMR in the Management of Chronic Coronary Syndrome. *Echocardiography (Mount Kisco, N.Y.)*.

[5] Vrints C, Andreotti F, Koskinas KC, Rossello X, Adamo M, Ainslie J (2024). 2024 ESC Guidelines for the management of chronic coronary syndromes. *European Heart Journal*.

[6] Neumann FJ, Sousa-Uva M, Ahlsson A, Alfonso F, Banning AP, Benedetto U (2019). 2018 ESC/EACTS Guidelines on myocardial revascularization. *European Heart Journal*.

[7] Escaned J, Berry C, De Bruyne B, Shabbir A, Collet C, Lee JM (2023). Applied coronary physiology for planning and guidance of percutaneous coronary interventions. A clinical consensus statement from the European Association of Percutaneous Cardiovascular Interventions (EAPCI) of the European Society of Cardiology. *EuroIntervention: Journal of EuroPCR in Collaboration with the Working Group on Interventional Cardiology of the European Society of Cardiology*.

[8] Johnson TW, Räber L, di Mario C, Bourantas C, Jia H, Mattesini A (2019). Clinical use of intracoronary imaging. Part 2: acute coronary syndromes, ambiguous coronary angiography findings, and guiding interventional decision-making: an expert consensus document of the European Association of Percutaneous Cardiovascular Interventions. *European Heart Journal*.

[9] Räber L, Mintz GS, Koskinas KC, Johnson TW, Holm NR, Onuma Y (2018). Clinical use of intracoronary imaging. Part 1: guidance and optimization of coronary interventions. An expert consensus document of the European Association of Percutaneous Cardiovascular Interventions. *European Heart Journal*.

[10] Mohr FW, Morice MC, Kappetein AP, Feldman TE, Ståhle E, Colombo A (2013). Coronary artery bypass graft surgery versus percutaneous coronary intervention in patients with three-vessel disease and left main coronary disease: 5-year follow-up of the randomised, clinical SYNTAX trial. *Lancet (London, England)*.

[11] Escaned J, Collet C, Ryan N, De Maria GL, Walsh S, Sabate M (2017). Clinical outcomes of state-of-the-art percutaneous coronary revascularization in patients with de novo three vessel disease: 1-year results of the SYNTAX II study. *European Heart Journal*.

[12] Kirtane AJ, Doshi D, Leon MB, Lasala JM, Ohman EM, O’Neill WW (2016). Treatment of Higher-Risk Patients With an Indication for Revascularization: Evolution Within the Field of Contemporary Percutaneous Coronary Intervention. *Circulation*.

[13] Sandhu A, McCoy LA, Negi SI, Hameed I, Atri P, Al’Aref SJ (2015). Use of mechanical circulatory support in patients undergoing percutaneous coronary intervention: insights from the National Cardiovascular Data Registry. *Circulation*.

[14] Dong Y, Xu Z, Dai XF, Chen LW, Lin ZQ (2024). Clinical Outcomes and Left Ventricular Functional Remodeling after Extracorporeal Membrane Oxygenation Assisted Percutaneous Coronary Intervention in Patients with Ischemic Cardiomyopathy: A Single-Center Retrospective Observational Study of 76 Cases. *Reviews in Cardiovascular Medicine*.

